# Heterometal nanoparticles from Ru-based molecular clusters covalently anchored onto functionalized carbon nanotubes and nanofibers

**DOI:** 10.3762/bjnano.6.133

**Published:** 2015-06-10

**Authors:** Deborah Vidick, Xiaoxing Ke, Michel Devillers, Claude Poleunis, Arnaud Delcorte, Pietro Moggi, Gustaaf Van Tendeloo, Sophie Hermans

**Affiliations:** 1 Institute of Condensed Matter and Nanosciences (IMCN), Université catholique de Louvain, Place Louis Pasteur 1/3, B-1348 Louvain-la-Neuve, Belgium; 2 EMAT (Electron Microscopy for Materials Science), University of Antwerp, Groenenborgerlaan 171, B2020 Antwerpen, Belgium; 3 Institute of Condensed Matter and Nanosciences (IMCN), Université catholique de Louvain, Croix du Sud 1, B-1348 Louvain-la-Neuve, Belgium,; 4 Dipartimento di Chimica, Università di Parma, Parco Area delle Scienze 17/A, 43124 Parma, Italy

**Keywords:** ammonia synthesis, cluster, nanofibers, nanoparticles, nanotubes

## Abstract

Heterometal clusters containing Ru and Au, Co and/or Pt are anchored onto carbon nanotubes and nanofibers functionalized with chelating phosphine groups. The cluster anchoring yield is related to the amount of phosphine groups available on the nanocarbon surface. The ligands of the anchored molecular species are then removed by gentle thermal treatment in order to form nanoparticles. In the case of Au-containing clusters, removal of gold atoms from the clusters and agglomeration leads to a bimodal distribution of nanoparticles at the nanocarbon surface. In the case of Ru–Pt species, anchoring occurs without reorganization through a ligand exchange mechanism. After thermal treatment, ultrasmall (1–3 nm) bimetal Ru–Pt nanoparticles are formed on the surface of the nanocarbons. Characterization by high resolution transmission electron microscopy (HRTEM) and high angle annular dark field scanning transmission electron microscopy (HAADF-STEM) confirms their bimetal nature on the nanoscale. The obtained bimetal nanoparticles supported on nanocarbon were tested as catalysts in ammonia synthesis and are shown to be active at low temperature and atmospheric pressure with very low Ru loading.

## Introduction

Metal nanoparticles (NPs) supported on nanoscopic forms of carbon (nanotubes, nanofibers) are an important class of nanostructured materials that find applications in a wide range of areas [1–5] due to the unique properties of the nanocarbons (conductivity, mechanical resistance, high surface area, etc.) combined with the size-dependent properties of the metal NPs. Due to the excellent electrical conductivity of carbon, NPs/nanocarbons are widely used in electrochemical devices where metal/carbon nanostructured materials can be used as superior electrodes [4]. Ultrasmall metal nanoparticles are desired in order to increase (electro-)catalytic activity by increasing the active metal surface. This is especially important in the case of precious metals. Moreover, bimetal nanoparticles supported on nanocarbons have attracted much interest since synergetic effects could enhance the global activity, as compared with pure metal.

In particular, Ru–Pt NPs supported on carbon nanotubes (CNT) (mostly multiwalled nanotubes (MWNT), or carbon nanofibers (CNF)) are well suited as anodes for direct methanol fuel cells (DMFC) [6–9], which hold much prospect as a portable energy source for mobile devices. The electrocatalytic activity of Pt–Ru/CNF [7] or Pt–Ru/MWNT [10] composite electrodes for methanol oxidation is found to be better than that of commercial Pt–Ru/C catalysts. The preparation methods for Pt–Ru/nanocarbon are varied and take inspiration from (i) electrochemistry (electrodeposition) [11–12], (ii) nanoparticle synthesis (polyol procedure) [13–14] or (iii) heterogeneous catalysis (impregnation/reduction). A fixed pH value during the polyol process has been found to influence the nanoparticle size, composition, and catalytic activity [15]. In addition, other effects have been studied, such as the presence of oxygenated groups on MWNTs introduced by acid pretreatment [16], the Pt to Ru atomic ratio [17–18], the lengths and diameters of the MWNTs used [19] and the type of CNT (single-, double- or multi-walled nanotubes) considered [20]. Bimetal Ru–Pt catalysts for the hydrogenation of cinnamaldehyde were prepared by impregnation of carbon nanotubes, graphite nanofibers (GNFs) and activated carbon (AC) for comparison [21]. It was found that their average particle size follows the sequence of GNF < MWNT ≈ SWNT < AC, but the activity and selectivity are sensitive to other factors such as porosity and surface chemistry of the carbon support [21–22]. Finally, alternative preparation methods have also been reported, including supercritical carbon dioxide [23–24], ultrasonic treatment [25–26] or H_2_ plasma treatment [27], for instance. Glucose sensors based on PtRu/MWNT have been elaborated as well [28]. These studies on Ru–Pt/MWNT materials give at best global EDX analyses [10–11,17] and XPS results [15]. However, these are both elemental analyses, and do not prove that both metals are mixed locally within each nanoparticle. In some other cases, powder XRD proved alloy formation [25], but this is only applicable for crystalline nanoparticles that are usually >3 nm [13,23,29].

In this paper, we explore the possibility of using molecular mixed-metal clusters as precursors for depositing heterometal nanoparticles on carbon nanotubes and nanofibers. The advantages of such precursors are that they present well-defined molecular structures and exchangeable ligands, the metal atoms are in the zero oxidation state and bimetal associations can be synthesized with a large degree of freedom. This ensures the intimate mixing of the metals when nucleating heterometal nanoparticles by thermal treatment. Because this treatment only requires removing the ligand sheath, ultrasmall, mixed-metal entities directly derived from the cluster cores might be expected. For that purpose, MWNTs and CNFs functionalized with chelating phosphine groups are used as supports. The clusters that were reacted with functionalized nanocarbon surfaces included: homometal Ru, hetero-bimetal Ru–M (M = Au, Pt, Co) and a hetero-trimetal Ru–Au–Pt species of general formula [Ru*_x_*M*_y_*C(CO)*_z_*L*_z_*] where M = Pt or Au and L = COD (1,5-cyclooctadiene) or PPh_3_ [30–36]. The letter M is used to designate the second metal present in the ruthenium-based bi(tri)metal cluster core. Only the Ru–Co cluster is negatively charged and hence presents a counterion, bis(triphenylphosphine)iminium (PPN^+^), giving the formula PPN[Ru_3_Co(CO)_13_]. It is known that all these clusters can exchange CO or COD ligands with phosphine ligands [37]. In addition, the negatively charged Ru–Co species can also interact with a support functionalized with positively charged ammonium groups (–NMe_3_^+^) [38]. The bimetal Ru–Pt association is justified by possible applications in fuel cells as described above, while Au is also considered in this study since Au NPs supported on nanocarbons can be used as sensors for the detection of gases or various life-related molecules such as glucose [4]. The amount of clusters that can be loaded onto a nanocarbon structure will be evaluated, as well as their behavior during the first steps of thermal treatment, which aims to gently remove the ligands and coalesce the metal cores to form ultrasmall heterometal nanoparticles supported on nanocarbons. It is generally believed that the clusters first lose their ligands, accompanied by an increased interaction with the surface, and then agglomerate with prolonged heat treatment [39–41]. This study is dedicated to the first stage of activation, i.e., denuded cluster cores before agglomeration, and in particular, their characterization at atomic resolution to prove their bimetal nature within individual nanoparticles. In order to test their potential application in catalysis, the carbon-supported nanoparticles are evaluated in ammonia synthesis, as a reference reaction with mature technology. The goal is not to optimize the catalytic performance but rather to demonstrate proof-of-principle activity in a well-known reaction.

The synthesis of ammonia from its constituting elements (N_2_ + 3H_2_) is one of the largest industrial processes based on heterogeneous catalysis [42]. Owing to the extreme inertness of N_2_, its chemical transformation commonly requires remarkably drastic conditions. Thus, even after extensive improvements, the industrial process of producing NH_3_ from N_2_ and H_2_ gas still requires very high pressures (15–35 MPa) and temperatures (400–550 °C) [43–44]. Therefore, the catalytic synthesis of ammonia under milder conditions has been a longstanding goal in catalysis. The early development of catalysts for ammonia synthesis was based on iron. Only Ru has been found to be a suitable alternative material and became the second generation catalyst for ammonia synthesis. While pure Ru is practically inactive, the addition of s-block metals (K, Cs, Ba) dramatically enhances the ammonia yield [45–47]. Generally, the highest catalytic activities are obtained with carbon supports, and particularly with partially graphitized carbons [48]. Promoted Ru catalysts are active but more expensive than iron, hence other alternatives such as cobalt were explored [49]. Zhenwei et al. have studied supported Ru–M (M = Fe, Co, Ni, Mo) with potassium as a promoter for ammonia synthesis [50]. They found that the Ru–Co catalysts have the highest activity, hence we incorporated this bimetal association in our study for comparison. Nanocarbon-supported catalysts have already been reported in the literature for this reaction, allowing for a benchmark for our systems. In this paper, electrochemical applications were not explored in order to avoid issues related to sample preparation and the presence of additives, binders, etc.

## Results and Discussion

### Cluster anchoring

**Figure 1:**
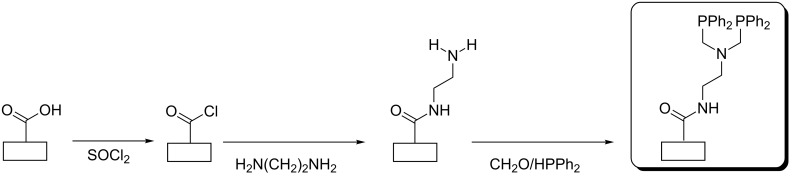
Schematic drawing to illustrate the functionalization of CNFs and MWNTs.

Phosphine-functionalized carbon nanofibers and nanotubes were prepared in several steps, as previously reported [38] and shown in Figure 1. We have also successfully applied this methodology to ordered mesoporous carbon [51]. Briefly, the number of oxygenated surface functional groups is increased first by HNO_3_ oxidation and quantified by XPS and elemental analysis. Then, the carboxylic acid groups are reacted with SOCl_2_, before coupling with ethylenediamine. Finally, the pendant amine functions are transformed into bidentate chelating phosphines by the combined action of formaldehyde and diphenylphosphine or into charged ammonium groups by quaternization [38,51]. The final supports, denoted as CNF–PPh_2_ (or CNF–NMe_3_^+^ in the case of ammonium groups) and MWNT–PPh_2_, are characterized by XPS, elemental analysis and nitrogen adsorption isotherms. XPS reveals the presence of the expected heteroatoms at each step of the functionalization strategy and indicates (from the ratios of atom % on the surface) that each reaction did not proceed with 100% yield and some unreacted sites remained from the previous step. The textural properties of the different supports were measured by nitrogen adsorption. The specific surface area (*S*_BET_) of oxidized carbon nanofibers (CNFox) was found to be 43 m^2^/g, 32 m^2^/g for CNF–PPh_2_, 29 m^2^/g for CNF–NMe_3_^+^, while much larger values of 325 m^2^/g for oxidized carbon nanotubes (MWCNTox) and 241 m^2^/g for MWCNT–PPh_2_ were found. Elemental analyses of the final CNF–PPh_2_ and MWNT–PPh_2_ supports reveal a P/C ratio (mass) equal to 0.005 and 0.013, corresponding to ≈1.5 and ≈0.45 anchoring sites per nm^2^, respectively.

Clusters **1** to **9**, comprised of Ru and Pt, Co and/or Au, were prepared as described in the Experimental section and are identified by their infrared ν_CO_ bands (Table S1 in Supporting Information File 1). In our previous related publications [38,51], these clusters were reacted with the functionalized ordered mesoporous carbon but not nanotubes and nanofibers. In order to prove the necessity of functionalizing the carbonaceous surfaces to obtain cluster loading, Ru_5_PtC(CO)_14_(COD) (**4**) and Ru_6_C(CO)_16_(Au{PPh_3_})_2_ (**5**) were selected as illustrative examples and contacted with pristine or oxidized (denoted as “ox”) CNF and MWNT. In all cases, the loading yield was experimentally determined. The loading yield is defined as the ratio between the amount of metal loaded onto the support (determined by ICP analysis of the solid) and the amount of metal introduced at the start (known). The solids are recovered by filtration thus any nonreacted cluster remains in solution and is not analyzed by ICP. For cluster **4**, there is no loading (0%) on pristine supports and CNFox but 37% loading is observed on MWNTox. In the case of **5**, there is no loading on pristine or oxidized supports (loading yield = 0%).

The analyzed results clearly show that the functionalization is important for an effective loading of clusters on the CNFs and MWNTs nanocarbons. Oxygen-containing groups were not functionalized because these organometallic clusters are not oxophilic. Phosphine functionalization is necessary to ensure anchoring. Given that the anchoring process happens by ligand exchange at the surface, the driving force is the chelating ability of the bidentate phosphine groups. Indeed, we previously carried out extensive studies with the same ligands on activated carbon and obtained formal proof of the ligand exchange in the starting cluster and binding through new M–P bonds at the surface. This proof was obtained by secondary ion mass spectrometry (SIMS) by comparison with non-functionalized carbon samples, but also by reacting model compounds in solution and crystallizing the products to solve their crystal structure, confirming the hypothesis [52].

**Figure 2:**
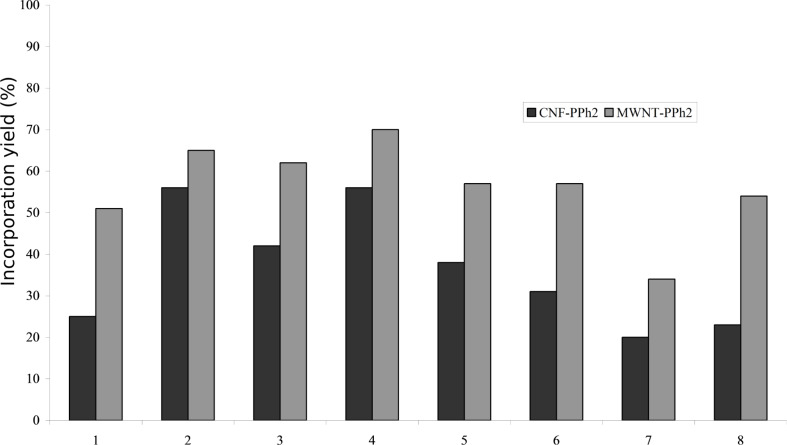
Anchoring yield (%) of clusters **1** to **8** on CNF–PPh_2_ and MWNT–PPh_2_.

Indeed, clusters **1–9** are anchored on the phosphine-functionalized nanocarbons by stirring in a mixture of toluene and dichloromethane. Toluene is chosen to maximize interactions with the carbon support and dichloromethane to ensure solubilization of the metal species. The total metal loading is determined in each case by Ru, Co, Pt and/or Au ICP analysis of the solid and the anchoring yield calculated as defined above (loading yield). The negatively charged cluster PPN[Ru_3_Co(CO)_13_] (**9**) is anchored with 28% yield on either a CNF–PPh_2_ or CNF–NMe_3_^+^ support. This is logical because it can either undergo a CO/phosphine ligand exchange (on CNF–PPh_2_) or form an ion pair by electrostatic interactions (on CNF–NMe_3_^+^). For species **1–8** (Figure 2), the anchoring yield is always better on MWNT–PPh_2_ than on CNF–PPh_2_. This result is explained by the fact that the amount of phosphine groups is higher on MWNT than on CNF (P/C determined by bulk elemental analysis, 0.013 and 0.005, respectively). The best anchoring yield is obtained for Ru_5_PtC(CO)_14_(COD) (**4**) while the lowest is obtained for Ru_4_C(CO)_12_(Au{PPh_3_})_2_ (**7**). This is due to the presence of an exchangeable COD ligand in the first case and the presence of the bulky Au/phosphine groups in the second case. Clusters containing gold in general lead to lower anchoring yields for the same reason. The presence of bulky phosphine groups causes steric repulsion and disfavors the cluster approach on the carbon surface. Moreover, Au presents a linear coordination geometry, hence, it binds to a terminal rather than chelating phosphine ligands. It is also known that Au atoms in clusters actually behave more as ligands than as part of the cluster core [53].

### SIMS characterization of anchored species

Cluster **4** anchored on CNF–PPh_2_, MWNTox and MWNT–PPh_2_ was characterized by secondary ion mass spectrometry (SIMS) after anchoring. It should be pointed out that the SIMS analysis was difficult because of undesired electrostatic interactions in the very narrow acceleration section (a few millimeters) between the grounded sample and the extraction lens of the spectrometer at 2 kV. These point effects can be explained by the elongated shape of the CNTs and CNFs. The molecular peak corresponding to the intact cluster was not observed. This could have been an indication of the fact that it is transformed by the anchoring process. However, both metals and some fragments are detected by SIMS at the surface, which indicates in this case that the cluster has not lost its molecular integrity. For example, on the positive mode, an isotopic peak centered at *m*/*z* 302 is observed in the SIMS spectra of Ru_5_PtC(CO)_14_(COD) (**4**) on CNF–PPh_2_ (Figure S1 in Supporting Information File 1) that corresponds to (PtPC_6_H_4_)^+^. The same fragment is also observed with the same cluster on the MWNT–PPh_2_ support. The PC_6_H_4_ moiety originates from the phosphine functions introduced beforehand on the support that have reacted with the cluster. As mentioned above, the surface ligand substitution mechanism was already studied in more detail by SIMS as well as model compounds in solution on activated carbon functionalized with the same chelating phosphines [52]. It was shown to take place effectively to give covalent anchoring of the cluster in a molecular form via metal–phosphorus bonds.

### Thermal activation

Thermogravimetric analysis of the pure clusters was carried out to determine the required activation temperature to remove the ligand layer surrounding the clusters (Table S2, Supporting Information File 1). The selected temperature was 300 °C for clusters **1** to **4**, 350 °C for clusters **5** to **8** and 400 °C for cluster **9**, for a duration of one hour, under nitrogen, in all cases. This corresponds to the removal of all ligands to produce naked clusters for species **1** to **4**. Clusters **5** to **9** loose their ligands more gradually over a wider temperature range (800–900 °C). However, they were not activated at such high temperatures to avoid sintering.

### XPS analysis

The Ru–M (M = Au, Pt) samples were characterized by XPS before and after thermal treatment to compare the various clusters with same metal nature but different composition. This study is thus not carried out for the unique Ru–Co species. The surface M/C ratios were determined (Table S3 in Supporting Information File 1). With CNF having a diameter of about 100 nm, XPS analysis is in this case clearly restricted to the surface (approximately 5 nm analysis depth for our experimental conditions). The XPS surface M/C ratios for clusters on CNF are always higher than the calculated values, indicating that the clusters are located on the external surface. The experimental XPS values obtained in the case of clusters anchored on MWNTs are very similar to the calculated values. Given the small diameter of MWNTs used here (≈10 nm) and the depth of XPS analysis (≈5 nm), we ascribe this observation to the fact that XPS behaves as a bulk elemental analysis technique for MWNTs. After thermal activation, most M/C ratio values decrease slightly, indicating that agglomeration occurred but to a limited extent. This process is more important for Au-based clusters (clusters **5** to **8**).

The Ru/M ratios were also determined by ICP and XPS (Table 1). The experimental ratios measured by ICP are relatively close to the theoretical values, as expected. There is a systematic error due to the known difficulties regarding Ru dissolution. Therefore, we conclude that clusters when broken down give fragments that all remain on the support but might segregate into separate entities. This correlates with the fact that when analyzing the loading solutions after filtration using infrared spectroscopy, we usually find the unreacted (unloaded) cluster in its intact molecular form. The experimental XPS values are quite different from the theoretical values, especially in the case of gold-containing clusters. This difference is explained by cluster fragmentation on the surface and confirms the above-mentioned conclusion.

**Table 1:** Comparison between the Ru/M ratios measured by ICP and XPS before and after thermal activation for clusters **3** to **8** on CNFs and MWNTs.

Cluster	Support	Ru/M calc.	Ru/M from ICP (mol)	Ru/M from XPS	
				Before activation	After activation

**3**	CNF–PPh_2_	6 (Pt)	4.99	3.25	2.50
	MWNT–PPh_2_		5.05	2.25	1.46
**4**	CNF–PPh_2_	5 (Pt)	4.37	3.16	2.43
	MWNT–PPh_2_		4.52	3.34	2.02
**5**	CNF–PPh_2_	3 (Au)	2.12	1.86	2.89
	MWNT–PPh_2_		2.61	1.32	2.08
**6**	CNF–PPh_2_	2.5 (Au)	2.01	1.36	4.72
	MWNT–PPh_2_		2.15	1.08	1.89
**7**	CNF–PPh_2_	2 (Au)	1.60	0.80	4.00
	MWNT–PPh_2_		1.91	0.72	1.56
**8**	CNF–PPh_2_	5 (Pt)	4.59	2.98	3.03
		2.5 (Au)	1.60	1.01	3.90
	MWNT–PPh_2_	5 (Pt)	4.40	0.78	0.41
		2.5 (Au)	1.54	0.45	0.75

### TEM investigations at low magnification

**Figure 3:**
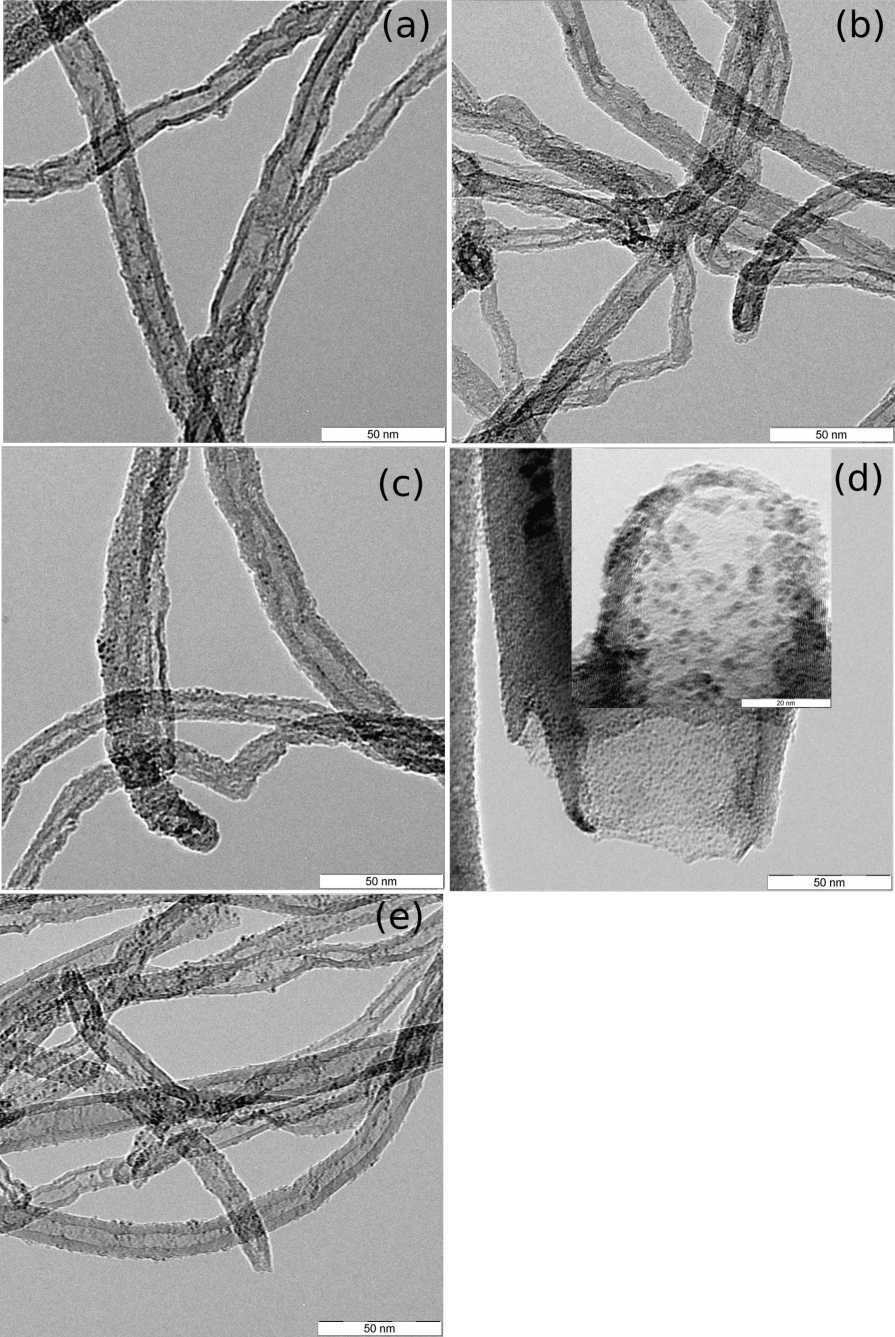
TEM images of clusters **1** to **3** on MWNT–PPh_2_ after thermal treatment (a) Ru_6_C(CO)_17_ (**1**), (b) Ru_5_C(CO)_15_ (**2**), (c) Ru_6_PtC(CO)_16_(COD) (**3**), and of cluster Ru_5_PtC(CO)_14_(COD) (**4**) after thermal treatment (d) on CNF–PPh_2_ (inset at higher magnification) and (e) on MWNT–PPh_2_.

Low magnification transmission electron microscopy (TEM) imaging is used to determine particle sizes after activation and to image their dispersion. TEM images of clusters **1** to **3** on MWNT–PPh_2_ (Figure 3a–c) reveal that nanoparticles with diameters of 1–3 nm are homogeneously dispersed on CNTs. Cluster **4** on MWNT–PPh_2_ (Figure 3f) or CNF–PPh_2_ (Figure 3d,e) exhibits particles with diameters <2 nm. Even if the final particles are ultrasmall, limited agglomeration occurred during thermal activation as the final particles sizes are slightly larger than the size of a single cluster (≈0.4 nm). The ligand substitution strategy for anchoring and stabilization of clusters is thus efficient in the case of Ru or Ru–Pt clusters.

TEM images of cluster **5** introduced on CNF–PPh_2_ (Figure S2a, Supporting Information File 1) show well-dispersed nanoparticles smaller than 3 nm, with accompanying presence of larger particles of 8–12 nm diameter as well. The same observations can be made with phosphine-functionalized nanotubes (Figure S2b Supporting Information File 1). These larger particles were analyzed by EDXS (Figure S2f, Supporting Information File 1), and it was revealed that these particles are constituted of Au only. Ru was found in the zones where the smallest particles (<3 nm) were found. The anchoring of these clusters on phosphine-functionalized supports causes its fragmentation. Similar results were previously observed on activated carbon [52], where it was shown that the cluster lost one Au atom during its anchoring on the support. During thermal treatment, these Au atoms tend to aggregate into larger particles.

The TEM images of the other Au-containing clusters **6** to **8** on MWNT–PPh_2_ (Figure S2c–e Supporting Information File 1) also show ultrasmall particles with diameters <3 nm and larger particles of different sizes depending on the cluster. For Ru_5_Au_2_C(CO)_14_(PPh_3_)_2_ (**6**), the size distribution of the large particles range between 15 and 20 nm, between 20 and 30 nm for Ru_4_Au_2_C(CO)_12_(PPh_3_)_2_ (**7**), and between 15 and 25 nm for Ru_5_PtAu_2_C(CO)_15_(PPh_3_)_2_ (**8**). The reactivity of the molecular cluster species with the functionalized surface thus has more influence on the size of nanoparticles obtained than the choice of the initial nanocarbon material.

### HRTEM and HAADF-STEM studies

**Figure 4:**
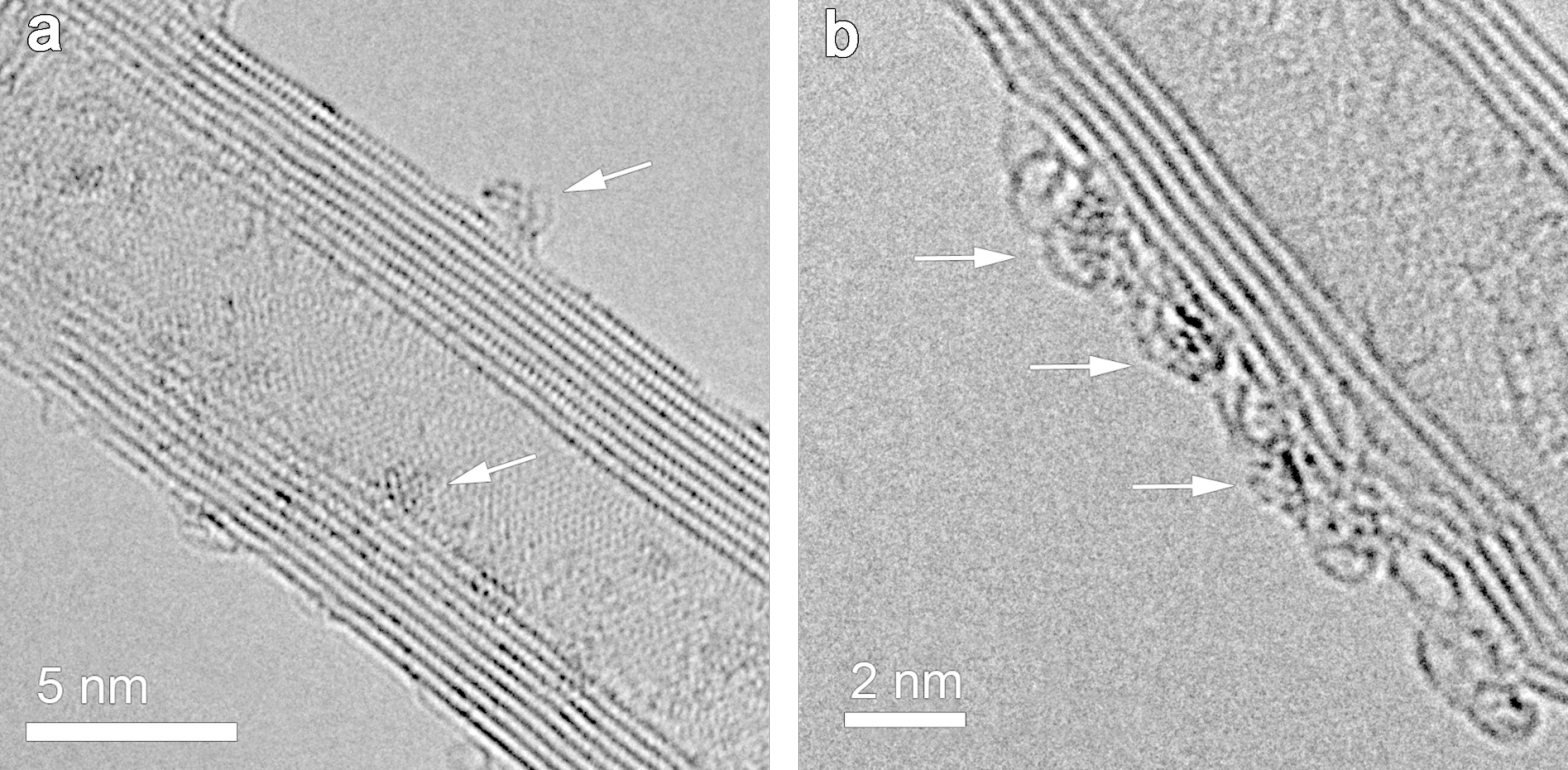
HRTEM images (Cs-corrected) of Ru–Pt/MWNT derived from Ru_5_PtC(CO)_14_(COD) (**4**) cluster. (a) Metal nanoparticles are indicated by arrows, (b) an amorphous layer can be seen around the nanoparticles at higher magnification.

In order to further study the structure of the supported Ru–Pt nanoparticles, the sample supported on MWNTs derived from Ru_5_PtC(CO)_14_(COD) (**4**) is characterized by high resolution transmission electron microscopy (HRTEM) and high angle annular dark field scanning transmission electron microscopy (HAADF-STEM) using aberration-corrected transmission electron microscopy (AC-TEM). HRTEM images of nanoparticles derived from cluster **4** reveal that they are not well crystallized (Figure 4a). This is due to the low temperature treatment applied for removing the ligands. In Figure 4b, an amorphous layer is observed around the nanoparticle, which is very likely due to the incomplete removal of ligands. The lack of crystallinity and amorphous layer coating might be detrimental for catalytic activity but could be overcome by higher temperature treatment. We have tested temperatures up to 1300 °C and these lead only to moderate agglomeration to reach particle sizes in the 10–15 nm range. This moderate agglomeration is due to stabilization by the anchoring arm.

**Figure 5:**
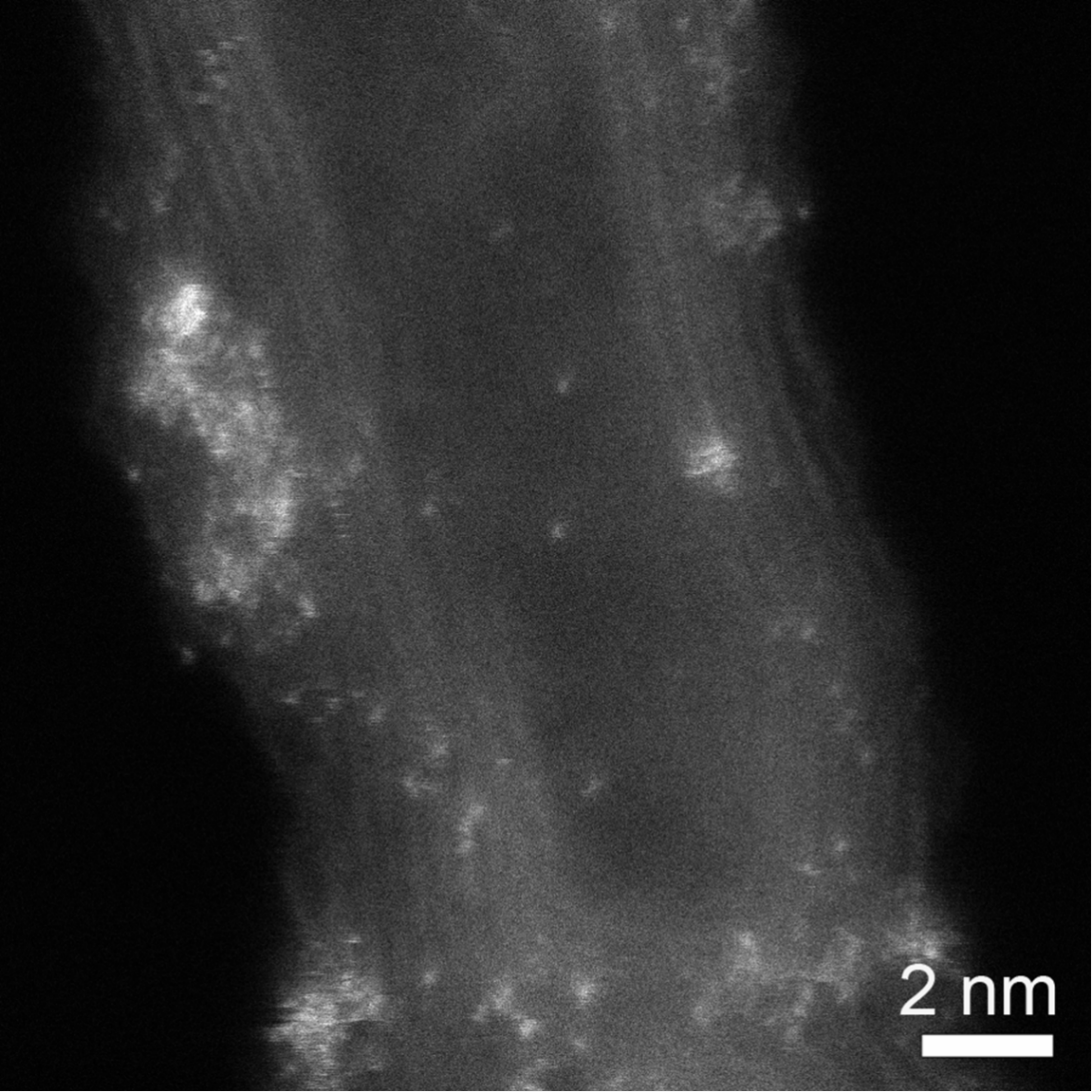
HAADF-STEM image of Ru–Pt/MWNT derived from Ru_5_PtC(CO)_14_(COD) (**4**).

In order to confirm the presence of the Ru/Pt bimetal nanoparticles, the sample was investigated using aberration-corrected HAADF-STEM and STEM energy dispersive X-ray (EDX) analysis. In HAADF-STEM, the intensity scales with the atomic number, *Z*. Therefore, the heavy elements in the nanoparticles, namely Pt and Ru, appear as bright contrast features. As shown in Figure 5, metal nanoparticles appear as a white contrast at atomic resolution. In addition, free standing individual atoms are visible on the CNT surface as well, which is likely to be caused by cluster collapse. This finding is important as it is generally believed that during thermal treatment the clusters lose their ligands before aggregating. Here we provide direct evidence for cluster fragmentation before agglomeration at the first stages of thermal activation. This was not expected as this Ru–Pt species was covalently anchored with molecular integrity before being submitted to activation.

**Figure 6:**
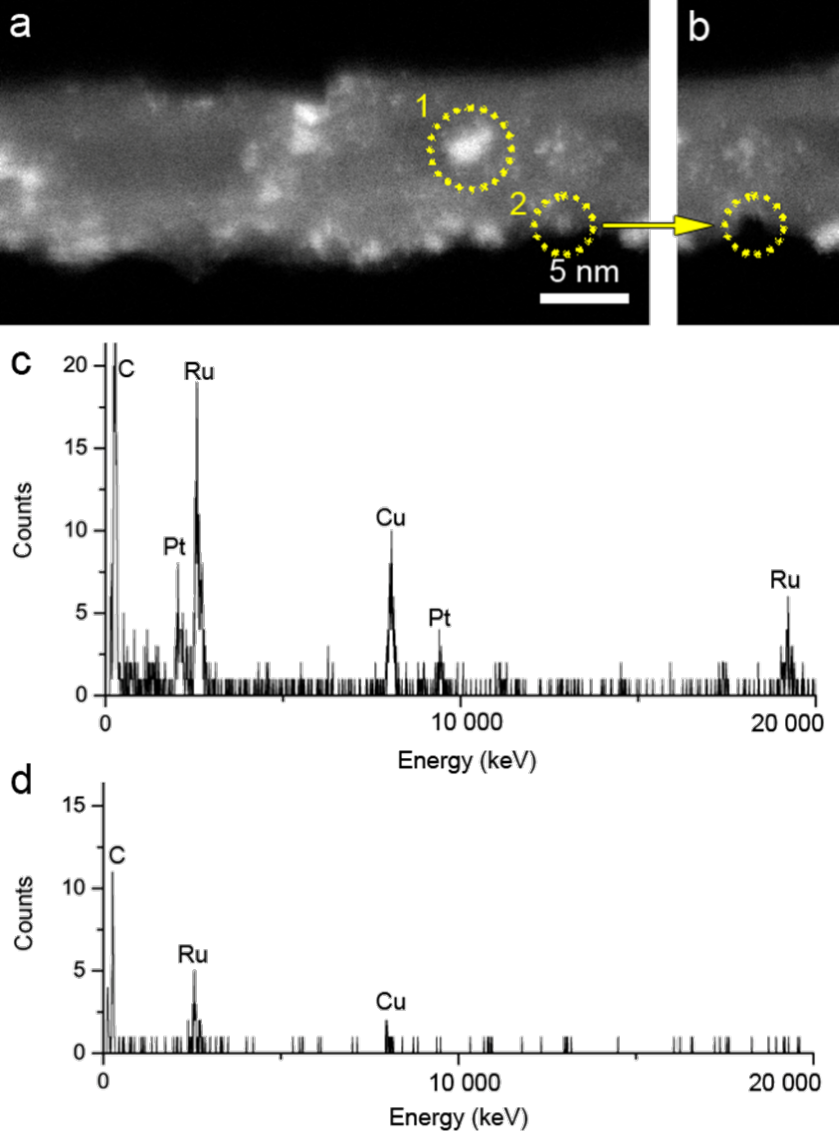
STEM-EDX of individual nanoclusters. (a) HAADF-STEM image of a nanotube with metal clusters. The spectra were collected from point 1 (larger particles ≈2–3 nm diameter particles) and point 2 (smaller particles <1nm). The corresponding spectra are shown as (c) point 1 and (d) point 2. (b) HAADF-STEM image of the same nanotube after spectra acquisition. The smaller particles where the spectrum in (d) is collected has been destroyed, see circled area. (c) EDX spectrum of a larger particle, where both Pt and Ru are present, but Ru has more counts. (d) EDX spectrum of a smaller particle, where only Ru is present. Low counts are probably due to the small volume of the ultrasmall particle.

In order to confirm the elemental composition of the ultrasmall nanoparticles, STEM-EDX spot analysis is performed over individual nanoparticles of diameter of approximately 2–3 nm, as shown in Figure 6a. The EDX spectrum taken from spot 1 confirms the presence of both Ru and Pt where Ru contributes more dose than Pt (Figure 6c). This is in agreement with a Ru/Pt 5:1 stoichiometry in the starting cluster. However, for ultrasmall nanoparticles, as indicated with spot 2 (approximately 1 nm in diameter), only Ru is detectable (Figure 6d). Due to the ultrasmall size of these nanoparticles, they are damaged by electron irradiation and disappear after the spectra acquisition (Figure 6b). This may explain the EDX point analysis of ultrasmall nanoparticles. The contribution of Pt, if present, is too small or too short-lived to be confirmed. These ultrasmall particles are most probably individual cluster cores, hence containing only one Pt atom in each nanoparticle.

The use of single source precursors was thus adequate for the formation of heterometal nanoparticles. The presence of both metals within a single ultrasmall, non-crystalline nanoparticle is proven, which is not amenable to XRD characterization.

### Catalysis

Despite the lack of crystallinity and amorphous layer coating observed by HRTEM, the obtained materials were tested as heterogeneous catalysts to demonstrate proof-of-principle activity. The reaction investigated was ammonia synthesis. This was carried out in a preliminary manner without any optimization of the reaction conditions. We have tested five catalysts based on Ru(–M) clusters prepared on phosphine- or ammonium-functionalized nanofibers (Table 2). The first synthesis step consisted of anchoring the Ru-based clusters on the functionalized nanofibers and subsequent thermal activation as described above. The percentage of Ru present on the carbon nanofibers at this stage was determined by ICP analysis (Table 2). In a second step, a promoter element, Cs, was added to the support by impregnation of the cesium oxalate compound followed by solvent evaporation (see Supporting Information File 1 for experimental details). The final activation under H_2_ was realized after determination of the experimental conditions by temperature programmed reduction (TPR). The samples were characterized by XPS and TEM after the catalytic test. Afterwards, small particles were still observed but with the concomitant presence of small agglomerates (5–10 nm) probably due to sintering during the catalytic test (see Figure S4 in Supporting Information File 1). The same TEM observations were made for the five catalysts.

**Table 2:** Description of catalysts prepared for ammonia synthesis.

Precursor	Support	Ru wt % after thermal activation

Ru_5_C(CO)_15_ (**2**)	CNF–PPh_2_	2.3
Ru_5_PtC(CO)_14_(COD) (**4**)	CNF–PPh_2_	3.0
Ru_5_C(CO)_14_(Au{PPh_3_})_2_ (**6**)	CNF–PPh_2_	2.6
Ru_5_PtC(CO)_15_(Au{PPh_3_})_2_ (**8**)	CNF–PPh_2_	2.3
PPN[Ru_3_Co(CO)_13_] (**9**)	CNF–NMe_3_^+^	1.7

The catalytic tests were realized at different temperatures and the results are presented in Supporting Information File 1. Table 3 summarizes the best results obtained for each catalyst. From these results, it can be concluded that the presence of Pt and particularly of Au is negative for the production of NH_3_. It is known that Pt easily dissociates H_2_ which would cover the Ru surface by H making it inaccessible for N_2_ dissociation. These results (ammonia synthesis rate, mmol NH_3_ h^−1^g^−1^ Ru) are as good as or better than some results obtained on inorganic oxides supports [54–55] but less (≈10×) than some results reported with carbon nanotubes and nanofibers in the literature [43,56–57] using different experimental catalytic testing conditions. This allows for a benchmark for our systems and shows that they are not yet highly performing. It is remarkable that significant activity was obtained although such a low temperature and at atmospheric pressure with very low Ru loading was used. The maximum Ru percentage anchored was between 2 and 3 wt %, while a higher percentage (5–10 %) of Ru was used in reported studies to guarantee high performance. In addition, it has been shown that ultrasmall particles (1–2 nm) are less active in ammonia synthesis than larger ones (3–4 nm) [58]. In our catalysts the majority of particles have a diameter of <2 nm. Finally, clusters **6**, **8** and **9** are characterized by a higher decomposition temperature. Therefore, the selected low activation temperature could adversely influence their catalytic activity together with residues from the functionalization arm (Cl, S, O, P) [59]. Thus, there is room for improvement of the catalytic performance by optimizing the thermal activation pretreatment.

**Table 3:** Ammonia production (%) and ammonia synthesis rate (mmol NH_3_ h^−1^g^−1^ cat or mmol NH_3_ h^−1^g^−1^ Ru) for catalysts prepared on functionalized carbon nanofibers (see Table 2).

	**2** (225 °C)	**4** (350 °C)	**6** (375 °C)	**8** (375 °C)	**9** (300 °C)

Ammonia synthesis rate (per g cat)	1.01	0.79	0.44	0.28	0.95
Ammonia synthesis rate (per g Ru)	43.98	26.23	16.86	12.22	55.72
Ammonia production (%)	0.48	0.41	0.21	0.13	0.44

## Conclusion

Samples of carbon nanofibers and nanotubes were functionalized with chelating phosphine groups. Ru-based clusters were covalently anchored onto the modified carbon surfaces by ligand exchange. The characterization of the materials demonstrated the molecular nature of grafted fragments. The cluster molecules were then denuded from their ligand layer by gentle thermolysis and coalesced into metal nanoparticles of very small sizes. In the case of gold-containing clusters, a bimodal size distribution of metal nanoparticles was obtained due to gold segregation from the cluster cores and strong aggregation. In the case of Ru–Pt precursors, heterometal nanoparticles of ultrasmall size were formed on the carbon fibers and MWNTs. We used a combination of HRTEM and STEM-EDX analysis to prove their bimetal nature on the scale of an individual nanoparticle. This demonstrated that mixed-metal clusters are suitable precursors for ultrasmall heterometal nanoparticles of controlled composition if their reactivity is taken into account. The obtained Ru–Pt/nanocarbon composites could find application in heterogeneous catalysis or as anodes for direct methanol fuel cells. After promotion with Cs, we showed that our Ru/C nanocomposites are indeed active in ammonia synthesis under very mild conditions.

## Experimental

The experimental strategy was, in general, very similar to previous, related studies [38,51]. All steps were carried out under N_2_ using standard Schlenk techniques. The solvents were distilled or degassed before use, stored under nitrogen on molecular sieves, and the obtained products were stored under Ar in a glovebox. The CNFs (PR-24-XT-PS-OX, named CNFox here for simplicity) were received from Applied Sciences, Inc. (USA) and MWNTs (of 90% C purity) were supplied by Nanocyl S.A. (Belgium). Non-carbon impurities were not found in the MWNT sample. The functionalized supports, CNF–PPh_2_, CNF–NMe_3_^+^, MWNTox and MWNT–PPh_2_, were prepared as described elsewhere [38]. All mentioned reactants were commercially available and used as received. Ru_3_(CO)_12_ was supplied by Alfa Aesar, while Au(PPh_3_)Cl, bis(triphenylphosphine)iminium chloride (PPNCl), Pt(COD)Cl_2_ (COD = 1,5-cyclooctadiene) and PPh_4_Cl were supplied by Aldrich. The experimental setup for the physicochemical methods of characterization is described Supporting Information File 1.

### Synthesis of clusters

All clusters were synthesized using a procedure outlined in the literature [30–31,33–36] except for the cluster Ru_6_C(CO)_16_(Au{PPh_3_})_2_ (**5**). This cluster was prepared by reacting 100 mg of (PPN)_2_[Ru_6_C(CO)_16_] (0.0466 mmol) with 2 equiv of Au(PPh_3_)Cl (46.1 mg, 0.0933 mmol) in 10 mL of dichloromethane. The mixture was stirred at room temperature for 1 h, then filtered and the solvent was removed under reduced pressure. The obtained powder was purified by column chromatography on silica (hexane/dichloromethane 50/50) to give **5** as a dark red powder. The different results of clusters characterization are summarized in Table S1 of Supporting Information File 1.

### Anchoring and activation of clusters

The cluster concentration involved in each experiment corresponds to a theoretical 5 wt % metal loading on the support directly after ligand removal. In a typical experiment, 8.7 mg of cluster **3**, for example, was stirred with 95 mg of CNF–PPh_2_ or MWNT–PPh_2_ in a 1:1 (v/v) mixture of toluene and dichloromethane (total volume of 20 mL) at room temperature for 5 days in the dark. The solid was filtered out, washed with dichloromethane and dried at room temperature under vacuum. The anchoring yield was measured by direct analysis of the metal in the solid samples by ICP-AES. The anchoring yield is defined as the ratio between the amount of metal determined by ICP analysis of the solid and the known amount of metal engaged at the start in solution.

The supported clusters were then submitted to thermal treatment in a tubular oven, STF 16/450 from Carbolite. The samples were placed into porcelain combustion boats and heated under N_2_ stream at 300 °C for 1 h for cluster **1** to **4**, at 350 °C for 1 h for cluster **5** to **8** and at 400 °C for cluster **9** (heating ramp rate: 100 °C/h).

## Supporting Information

File 1 Characterization of clusters, thermogravimetric analysis, tables with XPS results, SIMS spectra, TEM images and EDXS analysis, in addition to a description of physico-chemical methods of characterization (experimental details) and ammonia catalysis (synthesis, tests, results).
