# Supplementary material for: Heterometal nanoparticles from Ru-based molecular clusters covalently anchored onto functionalized carbon nanotubes and nanofibers
Source: Beilstein J Nanotechnol. 2015 Jun 10;6:1287–97. doi: 10.3762/bjnano.6.133 (PMC4505093; doi:10.3762/bjnano.6.133)
Supplement: File 1 — Characterization of clusters, thermogravimetric analysis, tables with XPS results, SIMS spectra, TEM images and EDXS analysis, in addition to a description of physico-chemical methods of characterization (experimental details) and ammonia catalysis (synthesis, tests, results). [file Beilstein_J_Nanotechnol-06-1287-s001.pdf]

Supporting Information
for

Heterometal nanoparticles from Ru-based molecular clusters covalently anchored onto functionalized carbon nanotubes and nanofibers

Deborah Vidick[1], Xiaoxing Ke[2], Michel Devillers[1], Claude Poleunis[3], Arnaud Delcorte[3], Pietro Moggi[4], Gustaaf Van Tendeloo[2] and Sophie Hermans[1,*,§]

Address: [1]Institute of Condensed Matter and Nanosciences (IMCN), Université catholique de Louvain, Place Louis Pasteur 1/3, B-1348 Louvain-la-Neuve, Belgium, [2]EMAT, University of Antwerp, Groenenborgerlaan 171, B2020 Antwerpen, Belgium, [3]Institute of Condensed Matter and Nanosciences (IMCN), Université catholique de Louvain, Croix du Sud 1, B-1348 Louvain-la-Neuve, Belgium, and [4]Dipartimento di Chimica, Università di Parma, Parco Area delle Scienze 17/A, 43124 Parma, Italy

Email: Sophie Hermans - Sophie.Hermans@uclouvain.be

*Corresponding author

[§]Fax: +32 10 47 23 30

Additional Experimental Data

Table S1: Characterization of clusters.

Cluster	Synthesis yield (%)	IR ν_{CO} (cm^{-1})[a,b]	Elemental analysis (%) (calculated values)	Ref.
$[Ru_6C(CO)_{17}]$ (**1**)	71	2068 (s), 2047 (s) (hexane)	Not determined	[30]
$[Ru_5C(CO)_{15}]$ (**2**)	89	2068 (s), 2034 (m), 2018 (w) (hexane)	Not determined	[30]
$[Ru_6PtC(CO)_{16}(COD)]$ (**3**)	31	2078 (m), 2035 (s), 2002 (m), 1975 (w), 1943 (w) 1821 (w)	C 20.59 (21.92), H 1.50 (0.88)	[31]
$[Ru_5PtC(CO)_{14}(COD)]$ (**4**)	67	2077 (m), 2050 (s), 2033 (s), 2011 (s), 1989 (w), 1966 (w) 1818 (w)	C 22.43 (22.78), H 1.10 (1.00)	[31]
$[Ru_6C(CO)_{16}(Au\{PPh_3\})_2]$ (**5**)	84	2067 (w), 2049 (s), 2017 (vs), 1965 (w), 1821 (m)	C 32.15 (32.07), H 1.64 (1.52)	[32]
$[Ru_5C(CO)_{14}(Au\{PPh_3\})_2]$ (**6**)	75	2065 (m), 2035 (m), 2020 (s), 2008 (s), 1975 (m), 1845 (w)	C 33.31 (33.51), H 1.64 (1.65)	[33, 34]
$[Ru_4C(CO)_{12}(Au\{PPh_3\})_2]$ (**7**)	67	2064 (vw), 2032 (s), 2022 (s), 2008 (w), 1990 (m), 1954 (w)	C 34.94 (35.22), H 1.71 (1.81)	[34]
$[Ru_5PtC(CO)_{15}(Au\{PPh_3\})_2]$ (**8**)	73	2068 (m), 2038 (s), 2015 (vs), 1968 (m), 1859 (m), 1834 (m)	C 30.55 (30.45), H 1.70 (1.47)	[35]
$(PPN)[Ru_3Co(CO)_{13}]$ (**9**)	57	2069 (w), 2019 (vs), 1824 (w), 1793 (w)	C 44.05 (46.53), H 2.57 (2.40), N 1.11 (1.11)	[36]

[a]Abbreviations: s = strong, m = medium, w = weak, br = broad and sh = shoulder

[b]Infrared spectra of the clusters were recorded in dichloromethane solution, unless otherwise stated.

Table S2: Thermogravimetric analysis under N_2.

Cluster	Weight loss (%)	Final decomposition temperature (°C)	Calculated weight loss (%)	Weight loss at selected activation temperature (%)[a]
$[Ru_6C(CO)_{17}]$ (**1**)	44	300	44<br>(= 17 CO)	44
$[Ru_5C(CO)_{15}]$ (**2**)	47	300	45<br>(= 15 CO)	47
$[Ru_6PtC(CO)_{16}(COD)]$ (**3**)	38	300	41<br>(= 16 CO + COD)	38
$[Ru_5PtC(CO)_{14}(COD)]$ (**4**)	38	300	41<br>(= 14 CO + COD)	38
$[Ru_6C(CO)_{16}(Au\{PPh_3\})_2]$ (**5**)	48	750	49<br>(= 16 CO + 2 PPh_3)	32
$[Ru_5C(CO)_{14}(Au\{PPh_3\})_2]$ (**6**)	49	900	50<br>(= 14 CO + 2 PPh_3)	26
$[Ru_4C(CO)_{12}(Au\{PPh_3\})_2]$ (**7**)	53	900	52<br>(= 12 CO + 2 PPh_3)	34
$[Ru_5PtC(CO)_{15}(Au\{PPh_3\})_2]$ (**8**)	45	800	46<br>(= 15 CO + 2 PPh_3)	30
$(PPN)[Ru_3Co(CO)_{13}]$ (**9**)	57	900	71<br>(= 13 CO + 1 PPN)	36

[a]300 °C for clusters **1** to **4**, 350 °C for clusters **5** to **8** and 400 °C for cluster **9**.

Table S3: Loading and XPS results for the incorporation of clusters 1 to 8 on CNFs and MWNTs.

Cluster	Support	Metal loading (wt %)[a]	XPS			
			M	M/C_{calc}[b] (×100)	M/C_{exp} before act. (×100)	M/C_{exp} after act. (×100)
**1**	CNF–PPh_2	2.0	Ru	0.25	0.85	0.97
	MWNT–PPh_2	4.2	Ru	0.53	0.36	0.30
**2**	CNF–PPh_2	2.3	Ru	0.28	1.45	1.14
	MWNT–PPh_2	3.0	Ru	0.37	0.36	0.24
**3**	CNF–PPh_2	2.2	Ru	0.19	0.83	0.69
			Pt	0.04	0.25	0.27
	MWNT–PPh_2	3.1	Ru	0.28	0.22	0.16
			Pt	0.06	0.10	0.10
**4**	CNF–PPh_2	2.8	Ru	0.24	1.08	0.94
			Pt	0.06	0.34	0.39
	MWNT–PPh_2	4.4	Ru	0.38	0.40	0.29
			Pt	0.09	0.12	0.14
**5**	CNF–PPh_2	2.2	Ru	0.14	0.73	0.49
			Au	0.07	0.39	0.17
	MWNT–PPh_2	2.8	Ru	0.20	0.15	0.09
			Au	0.08	0.11	0.04
**6**	CNF–PPh_2	2.6	Ru	0.17	0.61	0.62
			Au	0.08	0.45	0.13
	MWNT–PPh_2	2.8	Ru	0.18	0.14	0.09
			Au	0.09	0.12	0.05
**7**	CNF–PPh_2	1.1	Ru	0.06	0.18	0.21
			Au	0.04	0.13	0.05
	MWNT–PPh_2	2.0	Ru	0.12	0.06	0.04
			Au	0.07	0.05	0.03
**8**	CNF–PPh_2	2.3	Ru	0.11	0.47	0.33
			Pt	0.03	0.16	0.11
			Au	0.07	0.47	0.08
	MWNT–PPh_2	2.6	Ru	0.12	0.04	0.02
			Pt	0.03	0.05	0.06
			Au	0.02	0.09	0.03

[a]Metal loading calculated from incorporation yield determined by ICP analysis of metals in the solid samples.
[b]Calculated values are bulk molar ratios based on the experimental metal loadings. The amount of metal taken into consideration for the calculations corresponds to the amount incorporated on the support. The amount of C taken into consideration corresponds to the amount of support used by considering that it is only constituted of carbon.

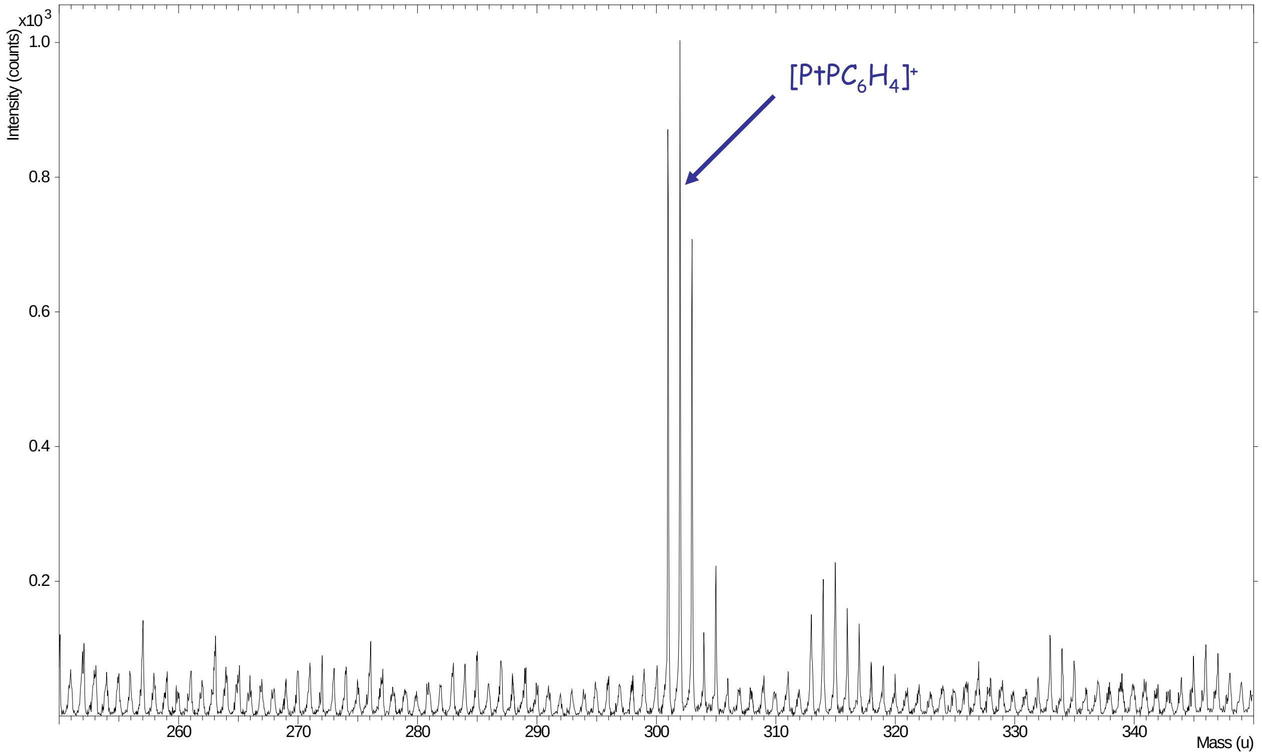

Figure S1: Positive SIMS spectrum of cluster **4** incorporated on CNF–PPh_2 (before thermal treatment).

(a)

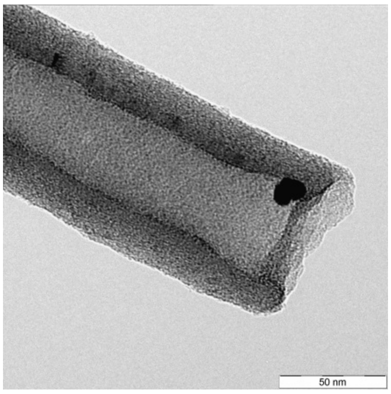

(b)

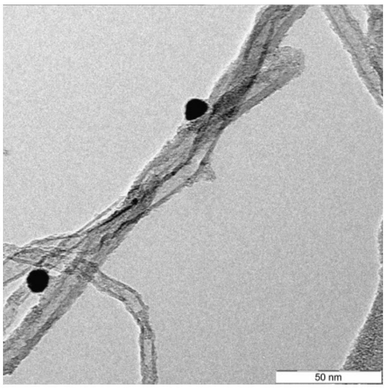

(c)

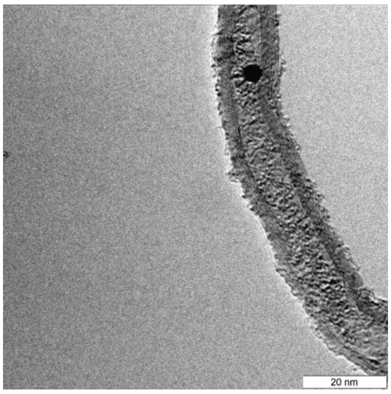

(d)

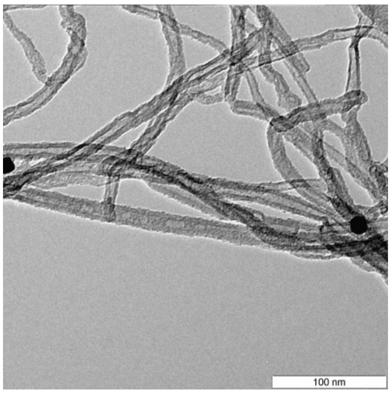

(e)

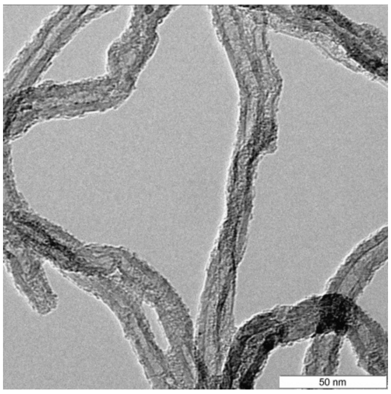

(f)

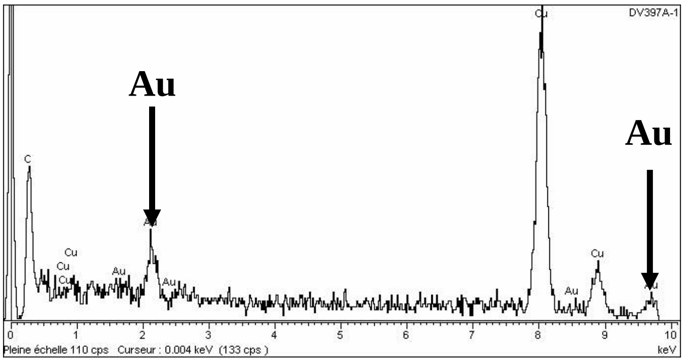

Figure S2: TEM images of cluster [$Ru_6C(CO)_{16}(Au\{PPh_3\})_2$] (**5**) after thermal treatment: (a) on CNF-PPh_2, (b) on MWNT-PPh_2. TEM images of cluster **6** to **8** on MWNT-PPh_2 after thermal treatment : (c) [$Ru_5Au_2C(CO)_{14}(PPh_3)_2$] (**6**) , (d) [$Ru_4Au_2C(CO)_{12}(PPh_3)_2$] (**7**) and (e) [$Ru_5PtAu_2C(CO)_{15}(PPh_3)_2$] (**8**). EDXS analysis of the large particles for cluster **5** on CNF-PPh_2 (f).

Physicochemical methods of characterization

The experimental strategies were in general very similar to our previous, related, studies [*J. Mater. Chem.*, **2013**, *1,* 2050-2063; *Catal. Today,* **2014**, *235,* 112-126]. Infrared spectra of the clusters were recorded in dichloromethane solution on a Bruker Equinox 55 spectrometer with a solution cell from Perkin Elmer.

The elemental analyses (C, H, N) were performed by the Analytical Chemistry service of University College London.

XPS analyses were performed on a Kratos Axis Ultra spectrometer (Kratos Analytical – Manchester – UK) equipped with a monochromatized aluminum X-ray source (powered at 10 mA and 15 kV). The sample powders were pressed into small stainless steel troughs mounted on a multi specimen holder. The pressure in the analysis chamber was about 10^{-6} Pa. The angle between the normal to the sample surface and the direction of photoelectrons collection was about 0°. Analyses were performed in the hybrid lens mode, the resulting analyzed area being 700 × 300 µm. The pass energy was set at 160 eV for the survey scan and 40 eV for narrow scans. In the latter conditions, the full width at half maximum (FWHM) of the Ag $3d_{5/2}$ peak of a standard silver sample was about 0.9 eV. Charge stabilization was achieved by using the Kratos Axis device. The following sequence of spectra was recorded: survey spectrum, C 1s, O 1s, N 1s, Cl 2p, S 2p, P 2p, Pt 4f (or Au 4f) and C 1s again to check for charge stability and the absence of degradation of the sample during the analyses. The C–(C,H) component of the C1s carbon peak has been fixed at 284.8 eV to set the binding energy scale. Spectra were decomposed with the CasaXPS program (Casa Software Ltd., UK) with a Gaussian/Lorentzian (70/30) product function and after subtraction of a linear baseline. Molar fractions were calculated using peak areas normalized on the basis of acquisition parameters, experimental sensitivity factors and transmission factors provided by the manufacturer. The constraints used for decomposition of p, d and f doublets are summarized in Table S4, with the FWHM ratio being equal to 1 in all cases. Given the overlap of the C 1s and Ru $3d_{5/2}$ peaks, these constraints were particularly important in order to quantify ruthenium. The following method was

used: a Gaussian/Lorentzian (85/15) was placed at the position of the Ru $3d_{5/2}$ peak, which is visible on the right-hand side (lower binding energy) of the C 1s peak. The contribution of the Ru $3d_{5/2}$ peak to subtract from the carbon component was calculated by reference to the Ru $3d_{5/2}$ peak by placing another Lorentzian/Gaussian (85/15) at 4.17 eV toward higher binding energy, and imposing an area ratio equal to 0.667. Because of this problem of overlap, the experimental error on the Ru surface atomic concentration is high and therefore the Ru/C surface ratios have to be taken with caution.

Table S4: Constraints for XPS results treatment.

Analytical peaks		Area ratio	Δ(B−A) (eV)
A	B	B/A	
Cl $2p_{3/2}$	Cl $2p_{1/2}$	0.500	1.6
S $2p_{3/2}$	S $2p_{1/2}$	0.500	1.18
P $2p_{3/2}$	P $2p_{1/2}$	0.500	0.84
Ru $3d_{5/2}$	Ru $3d_{3/2}$	0.667	4.17
Pt $4f_{7/2}$	Pt $4f_{5/2}$	0.750	3.33
Au $4f_{7/2}$	Au $4f_{5/2}$	0.750	3.67

Atomic emission measurements (ICP-AES) of platinum, gold and ruthenium were carried out on a ICAP6500 spectrophotometer from Thermo Scientific.

TOF-SIMS measurements were performed with an IONTOF V spectrometer. The sample was bombarded with pulsed Bi_3^+ ions (30 keV). The analyzed area used in this work was a square of 500 × 500 µm and the data acquisition time was 60 s. On insulating samples (such as the pure clusters), charge effects were compensated by means of an interlaced pulsed electron flood gun (E_k = 20 eV). With these parameters, the primary ion dose density was lower than 10^{11} Bi^+/cm^2. The powders were pressed with a spatula onto a double-sided silver adhesive sheet.

TGA analyses of the clusters were recorded on a SDT 2960 simultaneous DSC-TGA instrument from TA Instruments. These analyses were carried out with a heating ramp of 10 °C/min under N_2 flow (100 mL/min) with the samples placed into alumina containers.

Low magnification Transmission Electron Microscopy (TEM) images were obtained with a LEO 922 OMEGA energy filter TEM operated at 200 kV. The sample is prepared by suspending the solid samples in hexane which is then sonicated for 1 min. One drop of supernatant was then deposited on a holey carbon film supported on a copper grid, before being dried at room temperature overnight under vacuum prior to imaging.

High resolution TEM (HRTEM) studies were carried out by dispersing the sample powder in ethanol. The diluted suspension went through ultrasonic treatment for approximately 10 min to obtain a better dispersion, before being dropped onto a holey carbon film on a copper TEM grid. Aberration corrected HRTEM, high resolution high angle annular dark field scanning transmission electron microscopy (HAADF-STEM) and energy dispersive X-ray spectroscopy (STEM-EDX) experiments were carried out on a FEI Titan 50-80 “cube” microscope fitted with an aberration-corrector for the imaging lens and another for the probe forming lens as well as a monochromator and EDX detector, operated at 120kV in order to minimize knock-on damage to the supporting CNTs and CNFs. The monochromator was excited to extend the information limit down to approximately 1Å. HAADF-STEM was performed using a convergence semi-angle of 21.5 mrad.

Catalysis

i. Catalyst preparation

In a first step, the ruthenium-based precursors (**2**), (**4**), (**6**), (**8**) or (**9**) (5 wt % of Ru after ligands removal) and 850 mg of phosphine or ammonium functionalized nanofibers were introduced together in a 100 mL Schlenk flask with 1:1 mixture of toluene/dichloromethane (total volume = 80 mL). Then, the suspension was stirred at room temperature for 5 days in the dark under N_2 atmosphere. Finally, the solid was filtered out, washed with dichloromethane and dried at room temperature under vacuum. In the second step, the solid sample was pre-activated. It was placed into porcelain combustion boats and heated under N_2 stream at selected temperature during 1 h in a tubular oven. In the third step, the activated sample was reintroduced in a 100 mL Schlenk flask. Cesium oxalate was added to the solid (ratio 1:1 with respect to ruthenium, which was determined by ICP after activation under N_2) in a mixture of methanol/air-free water (9:1, total volume = 30 mL). The suspension was stirred at room temperature for 1 hour under N_2 atmosphere and then the solvent was evacuated in vacuo. The solid was dried overnight. The final activation was realized after charging the catalysts in the microreactor (400 °C, 4 h, under H_2). Table S5 summarizes the experimental conditions used for ruthenium-based catalysts synthesis.

Table S5: Experimental conditions used for the incorporation of ruthenium-based clusters on various supports.

Precursor	Precursor quantity (mg)	Support	Temperature of pre-activation
$[Ru_5C(CO)_{15}]$ (**2**) = Cat1	79	$CNF\text{-}PPh_2$	300 °C
$[Ru_5PtC(CO)_{14}(COD)]$ (**4**) = Cat2	102	$CNF\text{-}PPh_2$	300 °C
$[Ru_5C(CO)_{14}(Au\{PPh_3\})_2]$ (**6**) = Cat3	154	$CNF\text{-}PPh_2$	350 °C
$[Ru_5PtC(CO)_{14}(Au\{PPh_3\})_2]$ (**8**) = Cat4	172	$CNF\text{-}PPh_2$	350 °C
$(PPN)[Ru_3Co(CO)_{13}]$ (**9**) = Cat5	177	$CNF\text{-}NMe_3^+$	400 °C

ii. Catalyst testing

The catalytic tests were realized in a lab plant constructed for ammonia synthesis as presented in Figure S3. Two laboratory lines, N_2 and H_2 lines, were used with regulatory valves, to feed reagents. The flow rates were regulated with needle valves and were measured with a bubble flowmeter placed at the end of the plant. H_2 and N_2 from their lines went through a mixer where they were mixed together in the stoichiometric ratio (3:1) before going in the reactor. There, in the presence of a catalyst and at the controlled temperature, they reacted and then went through a series of gas collectors, the first two filled with a 0.1 mol/L solution of HCl and the last one with distilled water and a few drops of phenolphthalein as indicator to visualize any possible passage of NH_3. After this, the exit gas went into an aspirating nose connected to the global suction system. The ammonia produced was obtained by titration of liquid collected in the first two traps with NaOH 0.1 mol/L and phenolphthalein as indicator.

Reaction Conditions:

- Charge: 0.5 g of catalyst

- Pressure: atmospheric
- Temperature: from 250 to 400 °C
- H_2/N_2 (molar ratio) = 3:1
- Global Flow Rate: 40 mL/min at STP conditions

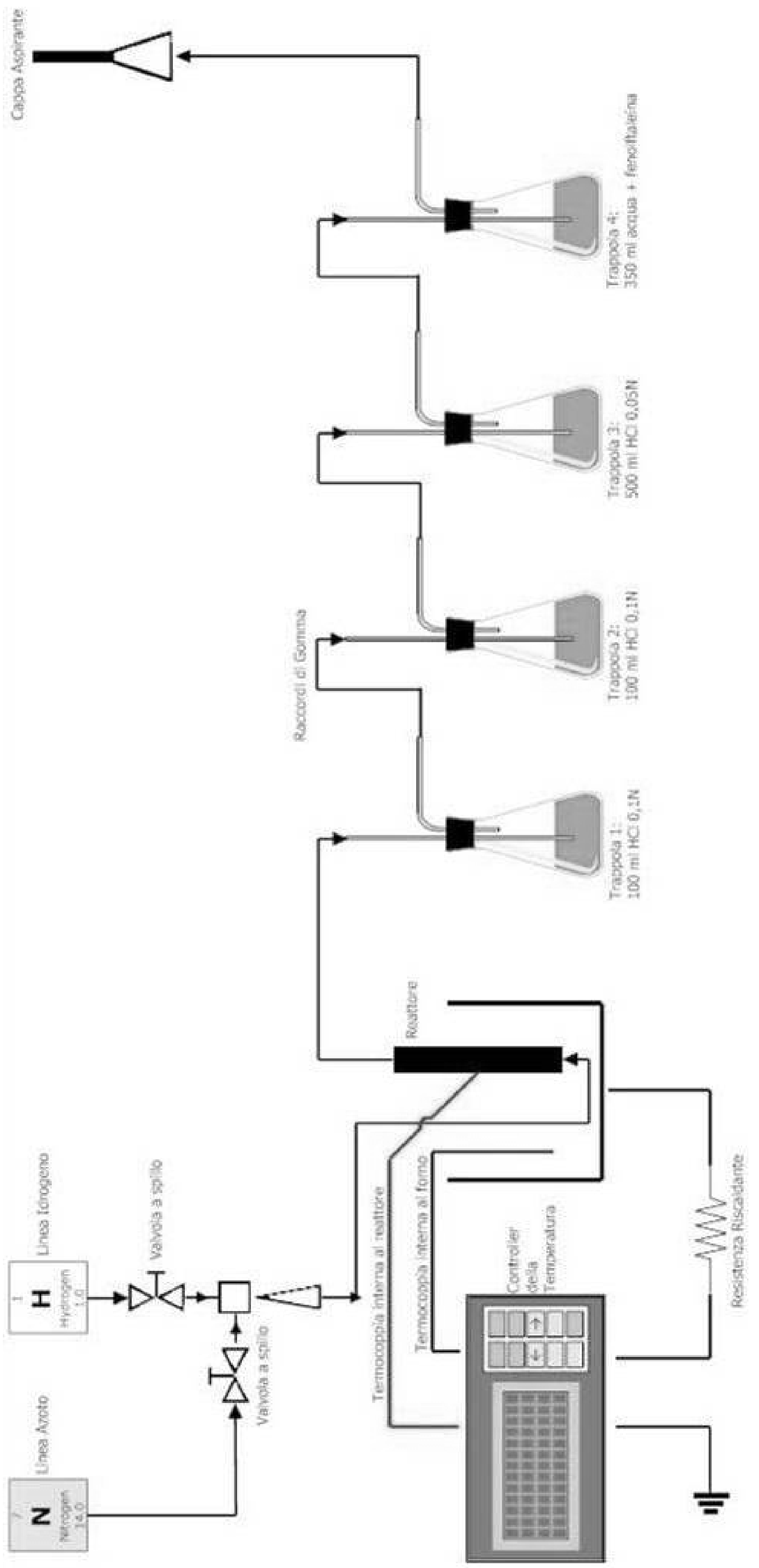

Figure S3: Laboratory plant for ammonia synthesis.

Ammonia synthesis: results

Table S6. Results in ammonia synthesis for Cs-promoted Ru_5/CNF-PPh_2 (Cat1)

	225°C	250°C	275°C	300°C
Time of test (h)	7	7	7	7
Weight of catalyst (g)	0.5042	0.5042	0.5042	0.5042
Total moles of NH_3 produced	0.0036	0.0026	0.0033	0.0023
Moles/h of NH_3 produced	0.0005	0.0004	0.0005	0.0003
mmol/h*g cata	1.0115	0.7367	0.9251	0.6446
Nitrogen feed (moles/h)	0.0268	0.0268	0.0268	0.0268
Hydrogen feed (moles/h)	0.0803	0.0803	0.0803	0.0803
Degree of conversion	0.0095	0.0069	0.0087	0.0060
NH_3 production (%)	0.4786	0.3481	0.4375	0.3044

Table S7. Results in ammonia synthesis for Cs-promoted Ru_5Pt/CNF-PPh_2 (Cat2)

	325°C	350°C	375°C	400°C
Time of test (h)	6	6	6	6
Weight of catalyst (g)	0.5506	0.5506	0.5506	0.5506
Total moles of NH_3 produced	0.0025	0.0026	0.0025	0.0024
Moles/h of NH_3 produced	0.0004	0.0004	0.0004	0.0004
mmol/h*g cata	0.7416	0.7870	0.7416	0.7113
Nitrogen feed (moles/h)	0.0268	0.0268	0.0268	0.0268
Hydrogen feed (moles/h)	0.0803	0.0803	0.0803	0.0803
Degree of conversion	0.0076	0.0081	0.0076	0.0073
NH_3 production (%)	0.3828	0.4063	0.3828	0.3671

Table S8. Results in ammonia synthesis for Cs-promoted Ru_5Au_2/CNF-PPh_2 (Cat3)

	350°C	375°C	400°C
Time of test (h)	5	5	5
Weight of catalyst (g)	0.5072	0.5072	0.5072
Total moles of NH_3 produced	0.0009	0.0011	0.0006
Moles/h of NH_3 produced	0.0002	0.0002	0.0001
mmol/h*g cata	0.3687	0.4383	0.2354
Nitrogen feed (moles/h)	0.0268	0.0268	0.0268
Hydrogen feed (moles/h)	0.0803	0.0803	0.0803
Degree of conversion	0.0035	0.0042	0.0022
NH_3 production (%)	0.1749	0.2083	0.1116

Table S9. Results in ammonia synthesis for Cs-promoted Ru_5PtAu_2/CNF-PPh_2 (Cat4)

	300°C	350°C	375°C	400°C
Time of test (h)	5	5	5	5
Weight of catalyst (g)	0.4980	0.4980	0.4980	0.4980
Total moles of NH_3 produced	0.0005	0.0006	0.0007	0.0005
Moles/h of NH_3 produced	0.0001	0.0001	0.0001	0.0001
mmol/h*g cata	0.2108	0.2490	0.2811	0.1867
Nitrogen feed (moles/h)	0.0268	0.0268	0.0268	0.0268
Hydrogen feed (moles/h)	0.0803	0.0803	0.0803	0.0803
Degree of conversion	0.0020	0.0023	0.0026	0.0017
NH_3 production (%)	0.0982	0.1159	0.1309	0.0869

Table S10. Results in ammonia synthesis for Cs-promoted Ru_3Co^-/CNF-NMe_3^+ (Cat5)

	300°C	350°C	375°C
Time of test (h)	5	5	5
Weight of catalyst (g)	0.4962	0.4962	0.4962
Total moles of NH_3 produced	0.0024	0.0004	0
Moles/h of NH_3 produced	0.0005	0.0001	0
mmol/h*g cata	0.9472	0.1532	0
Nitrogen feed (moles/h)	0.0268	0.0268	0.0268
Hydrogen feed (moles/h)	0.0803	0.0803	0.0803
Degree of conversion	0.0088	0.0014	0
NH_3 production (%)	0.4409	0.0710	0

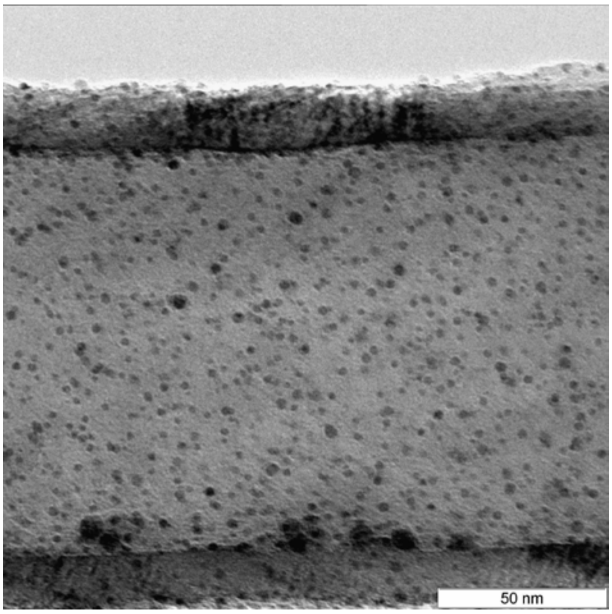

Figure S4: TEM images of Cata 1 (Ru_5/CNF-PPh_2) after test.
